# Unleashing the Potential: First‐in‐Human Evaluation of Automatic Robotic‐Assisted Endovascular Aortic Repair for Standardized Therapies

**DOI:** 10.1002/mco2.70489

**Published:** 2025-11-18

**Authors:** Bowen Liang, Chao Song, Shibo Xia, Wenying Guo, Longtu Zhu, Kundong Wang, Qingsheng Lu

**Affiliations:** ^1^ Department of Vascular Surgery, Shanghai Changhai Hospital Naval Medical University Shanghai China; ^2^ Department of Instrument Science and Engineering Shanghai Jiao Tong University Shanghai China

**Keywords:** automatic, endovascular aortic repair, surgical algorithm, surgical robot

## Abstract

Endovascular surgical robots have advanced vascular surgery through the integration of automatic programs for complex interventions. However, current systems still lack full procedural automation capabilities. This single‐center single‐arm study explores the feasibility and safety of automatic robotic‐assisted endovascular aortic repair (EVAR) using a novel surgical algorithm with in vitro and in vivo experiments. The EVAR process was deconstructed into surgical steps and programmed into an endovascular surgical robot, which executed the steps automatically based on parameters derived from image processing software. In vitro experiments using 3D‐printed vascular models demonstrated millimeter‐level precision, with reduced operation time, fluoroscopy time, and radiation exposure compared to manual robotic control. In vivo evaluations in four patients with abdominal aortic aneurysms achieved 100% technical and clinical success, with no major adverse events. Operation time averaged 110 ± 47 min, fluoroscopy time was 19 ± 6 min, and patient‐side radiation exposure was 1251 ± 389 mGy. Surgeon‐side radiation exposure was 4 ± 1 mGy. The results indicate that automatic robotic‐assisted EVAR can be performed with acceptable accuracy and safety to provide standardized therapies, shorten operation time, and reduce radiation exposure of patients.

## Graphical abstract

EVAR was deconstructed into surgical algorithms consisting of various parameters, which were obtained using image processing software and then inputted into the surgical robot. The endovascular surgical robot is programmed to automatically execute the surgical algorithm.

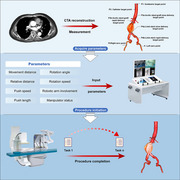

## 1 Introduction

Since its successful application in cardiovascular intervention [1, 2], the endovascular surgical robot has achieved significant advancements in percutaneous coronary intervention [3, 4, 5], peripheral vascular intervention [6, 7, 8], and neurointervention [9, 10, 11], thus showcasing its immense potential for conducting endovascular surgeries across diverse vascular anatomies. These advancements have not only improved procedural precision but also reduced operator radiation exposure and physical fatigue [12].

Within this evolving landscape, surgical automation represents a pivotal frontier in robotic surgery [13]. Current clinical practice reveals substantial inter‐operator and inter‐hospital variability in endovascular repair outcomes, with studies reporting suboptimal outcomes in low‐volume hospitals and surgeons [14], underscoring the urgent need for procedural standardization. Automation promises to standardize surgical tasks and minimize human variability. While endovascular surgical robots possess inherent advantages for automation—leveraging the predictable, tubular nature of vascular anatomy and modularizing complex maneuvers into fundamental robotic motions including advancement, retraction, and rotation [15], efforts to automate the entire procedural workflow remain scarce [16]. Although the Sensei X robotic system was used in EVAR procedures as early as 2009 and the Magellan robotic system was applied in fenestrated endovascular aneurysm repair (FEVAR) in 2013, the use of these robotic systems has been limited to cannulation of the contralateral limb of bifurcated stent grafts or target vessels [17, 18]. The recent integration of automatic functions into the CorPath GRX system has enabled more efficient guidewire manipulation during vascular interventions, although their application remains narrowly confined to this specific task [19]. Thus, the scope of executable robotic operations and the comprehensiveness of the automatic functionality remain inadequate. The inability of current robotic systems to support comprehensive automation is largely attributable to inherent limitations in their design, most notably in the engineering of endovascular device delivery components. Most platforms exhibit poor compatibility with standard off‐the‐shelf endovascular devices and lack the dexterity required for multi‐device coordination—critical capabilities for executing complex, multi‐step procedures such as EVAR. These inherent limitations in device adaptability and operational flexibility have significantly impeded the advancement of comprehensive surgical automation.

**FIGURE 1:**
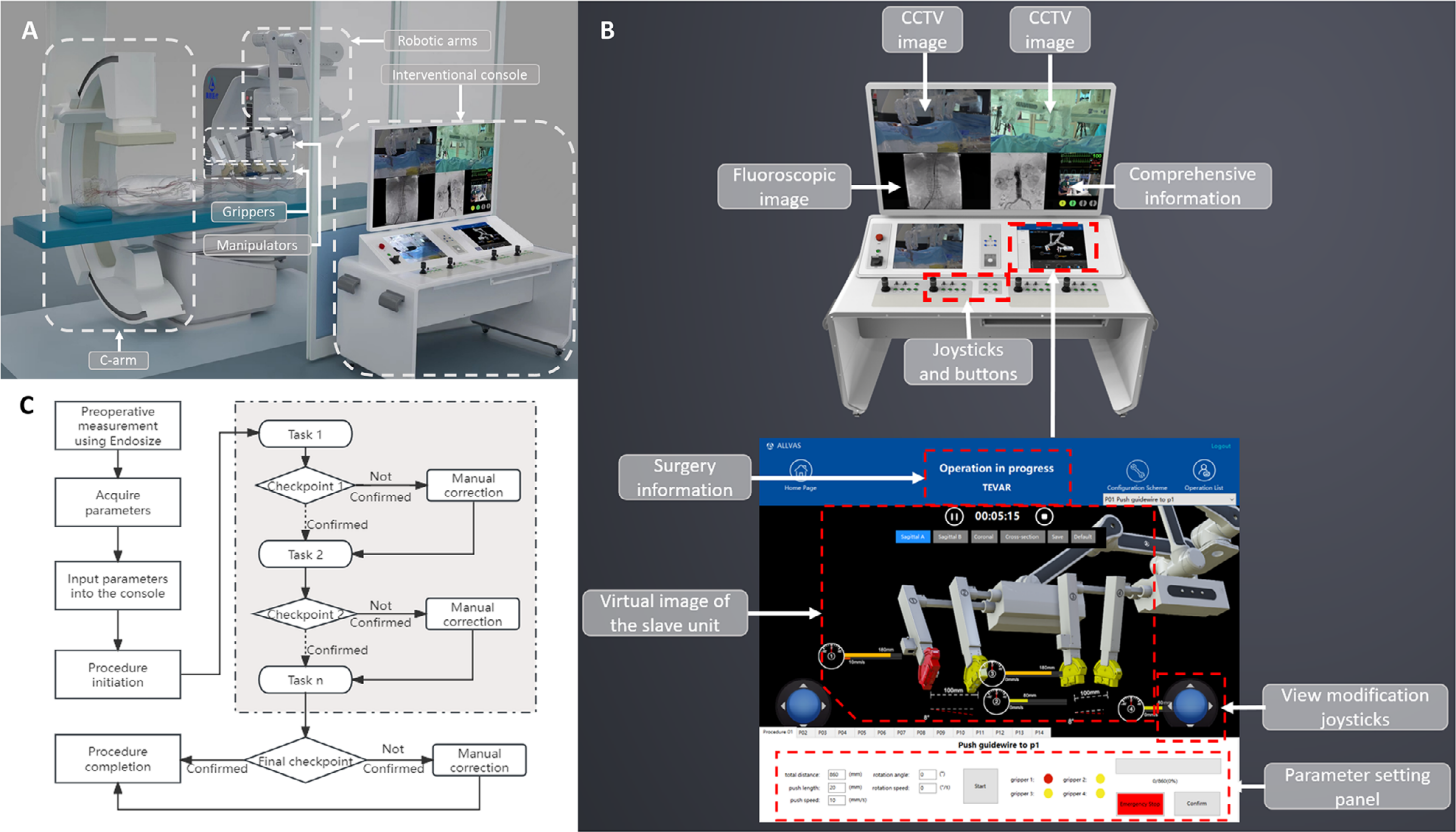
Composition and working principle of the endovascular surgical robot. (A) Overview of the endovascular surgical robot. During a procedure, the surgeon sits in front of the interventional console behind the lead shield, controlling the slave unit remotely. (B) Display and functions of the interventional console. (C) Flowchart of the execution of automatic robotic‐assisted endovascular procedure.

In response to these challenges, we developed a novel robotic platform (Figure 1) for endovascular procedures [20, 21], designed to be fully compatible with conventional endovascular devices and capable of performing the full spectrum of procedural maneuvers from device navigation and positioning to stent‐graft deployment. Building upon this system, we designed and implemented a suite of automatic surgical algorithms tailored for EVAR and assessed their feasibility and safety through in vitro and in vivo evaluations. This work represents a significant step toward clinical translation of automatic endovascular robotics, offering a scalable framework for future integration with real‐time imaging, machine learning, and closed‐loop control systems in endovascular surgery.

## 2 Results

### 2.1 Patient Characteristics

**FIGURE 2:**
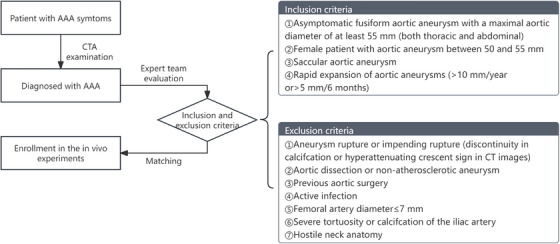
Patient enrollment flowchart.

This study introduced an automatic surgical algorithm designed for the endovascular surgical robot and carried out both in vitro and in vivo experiments to assess its feasibility and safety. Four patients with infrarenal abdominal aortic aneurysms were enrolled in the in vivo study after comprehensive assessment performed by an expert team comprised of vascular surgeons, anesthetist, radiologist, and technical engineers (see Figure 2). The demographic characteristics of the four patients enrolled in this study are presented in Table 1. The average age of the patients was 68 years, and two were men. All patients had fusiform aneurysms with a mean maximal aortic diameter of 57.9 ± 4.5 mm. None exhibited hostile neck anatomy (defined as the presence of at least one of the following: neck length <15 mm, neck diameter >28 mm, angulation >60°, mural thrombus or calcifications, or conical aortic neck).

**TABLE 1:** Demographic characteristics of four additional AAA patients involved.

Items	Patient 1	Patient 2	Patient 3	Patient 4	Mean ± SD, *n* (%)
Age (years)	74	61	71	65	68 ± 5
Male sex, *n* (%)	Male	Male	Female	Female	2 (50)
Comorbidity					
Hypertension, *n* (%)	Y	Y	Y	Y	4 (100)
Diabetes mellitus, *n* (%)	Y	N	Y	N	2 (50)
Hyperlipidemia, *n* (%)	Y	Y	N	N	2 (50)
COPD, *n* (%)	N	N	N	N	0 (0)
Peripheral vascular disease, *n* (%)	N	N	N	N	0 (0)
Past history					
Prior myocardial infarction, *n* (%)	N	N	N	N	0 (0)
Prior stroke or TIA, *n* (%)	N	N	N	N	0 (0)
Aneurysm features					
Location	Infrarenal	Infrarenal	Infrarenal	Infrarenal	—
Morphology	Fusiform	Fusiform	Fusiform	Fusiform	—
Maximal diameter of aorta (mm)	59.3	63.8	59.3	55.2	57.9 ± 4.5
Hostile neck^a^, *n* (%)	N	N	N	N	0 (0)
Neck length (mm)	22.8	18.3	20.9	34.8	28.3 ± 11.2
Neck diameter (mm)	26.4	29.7	26.4	27.1	27.0 ± 1.6
Neck angulation^b^ (°)	2.1	30.4	22.5	16.4	17.8 ± 10.4
Iliac access diameter	8.2	8.9	7.7	8.3	8.3 ± 0.4

^a^ A hostile neck was defined as the presence of at least one of the following: neck length <15 mm, neck diameter >28 mm, angulation >60°, mural thrombus/calcifications, and conical aortic neck.

^b^ Neck angulation refers to *α* angle, defined as the angle formed between the flow axis of the suprarenal aorta and the flow axis of the AAA neck.

### 2.2 Design of the Endovascular Surgical Robot

The endovascular surgical robot consists of two major parts: a slave unit and a master unit (see Figure 1A). The slave unit has two groups of independent mechanical arms with three degrees of freedom. Each arm has two manipulators controlling different kinds of endovascular devices, and each manipulator has one gripper. Previous research has already introduced the mechanical structure of manipulators and the principle of adjusting the clamping force adaptively [22]. The double V‐shaped grippers can clamp the thinnest 0.36 mm (0.014 inch) guidewire and the thickest 40 mm stent‐graft system, making them compatible with market‐leading devices. The master unit is an interventional console, which is the central processing unit of the whole robotic system (see Figure 1B). The interventional console consists of switch buttons, action buttons, holding buttons, action enabling buttons, an emergency button, joysticks, two monitors and a screen. Buttons and joysticks are used to control the movement of the slave unit. The real‐time virtual image of the slave unit, the basic surgical information, the surgical steps, and other information are available on the monitors. The dashboards in the real‐time virtual image of the slave unit specifically display the rotating angle of the Manipulator 1 (M1, highlighted in red color), while the upper portion of the progress bars signifies the length of the manipulators extending within their maximum range. The lower portion of the progress bar denotes the speed of the manipulator's movement. Additionally, spacing markers located at the bottom of the area indicate the distances between M1 and M2, as well as M3 and M4, while angle markers represent the inclination angle of the robotic arm axis in relation to the ground plane. In addition, the surgical robot can be controlled by the touchable parameter setting area at the bottom of the monitor. Fluoroscopic images, vital signs, CCTV pictures and states of manipulators are displayed on the screen above the console, which can be visualized at a closer distance.

In the process of endovascular device delivery, every two manipulators of each arm control one kind of device. Manipulators could be adjusted to a coaxial state for over‐the‐wire devices or a noncoaxial state for rapid‐exchange devices. An action unit of each manipulator with grippers can be customized as an appointed combination of basic actions such as advancement and retraction so that compound maneuvers such as stent‐graft deployment can be achieved. The speed and distance of each action unit are alterable and programmable. There are four action modes of the main V‐shaped gripper: loosening, holding, clamping and continuous rotation. Loosening keeps the endovascular device in the space between the double V‐shaped grippers, but the device is not in close contact with the grippers. Holding restricts the endovascular device to the space between the double V‐shaped grippers but does not restrict its axial movement. Clamping maintains the endovascular device in a locked state, making it completely follow the movement of the manipulator to advance, retract, and rotate. Continuous rotation is helpful in specific actions, such as arterial navigation [21] and stent‐graft deployment.

### 2.3 Deconstruction and Programming of Surgical Steps

EVAR processes were deconstructed into several surgical steps that formed a surgical algorithm (visualized in the form of tables, see Table S1). Each step was determined by several parameters, including distance, speed, angle, and status of robotic arms and manipulators. The robotic arms and manipulators were programmed to complete each step automatically after inputting these parameters in the parameter setting panel. The parameters were set by surgeons according to preoperative planning.

The operating mode of the surgical robot was a series connection of multiple automatic surgical steps, similar to an assembly line. There was a human‐controlled checkpoint between each step to ensure safety. After completing each surgical step, the surgeon clicked the confirm button on the monitor to proceed to the next surgical step. The endovascular surgical robot could execute each step continuously, but the confirmation part was introduced due to ethical and safety requirements. The robot could be overridden by the surgeon to maximize the safety of manipulation and return to the automatic mode again.

Execution of automatic surgical steps relied on preoperative planning. We calculated the target point and movement distance of the devices in each step through preoperative planning, and program the movements of the robotic arms, manipulators and grippers to achieve the established movement goals. The surgical robot itself had force feedback, which could be regarded as a safety mechanism for surgery to avoid the risk of iatrogenic injury. When the feedback force exceeds the 2N threshold—a value carefully determined to ensure both the smooth advancement of endovascular devices and the prevention of vascular wall perforation [23, 24]—the surgical robot will halt its automatic procedures, allowing the surgeon to assume control. In addition, there were physical and virtual emergency stop buttons on the console (see Figure 1B), which can stop the operation of the surgical robot in an emergency. The flowchart of the procedural execution is shown in Figure 1C.

### 2.4 Preoperative Planning for Automatic Procedure

**FIGURE 3:**
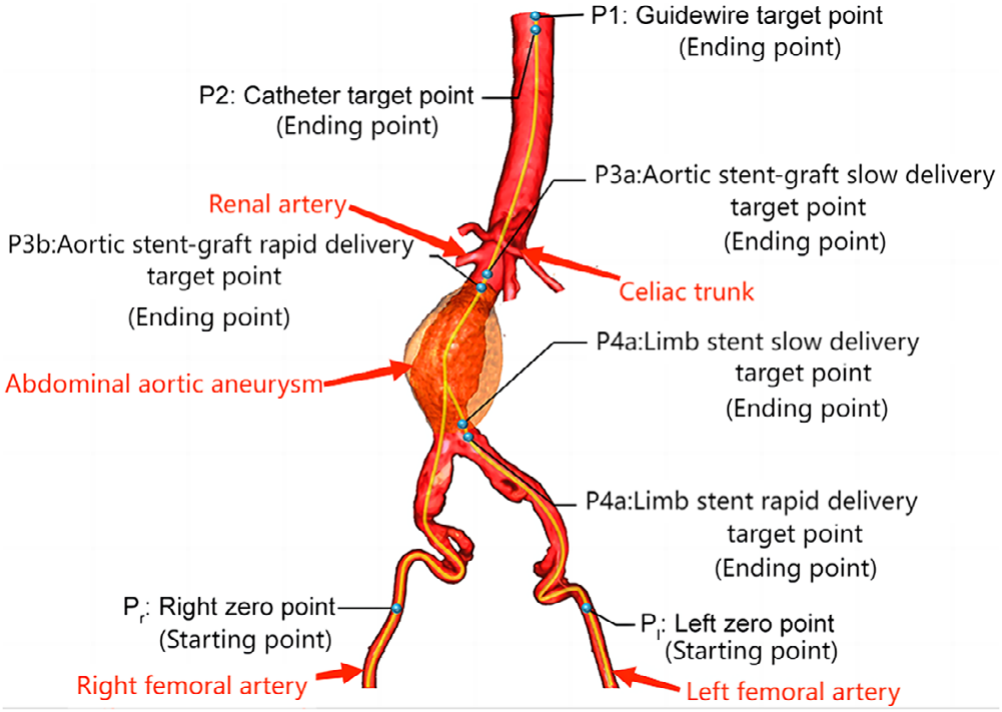
Preoperative planning for EVAR with Endosize. The central lines and points are shown in this figure. The movement distance of wires is from P_l_ to P1. The movement distance of catheters is from P_r_ to P2. The movement distance of the abdominal aortic stent graft is from P_r_ to P3a. The movement distance of the limb stent is from P_l_ to P4a.

Preoperative planning was composed of four parts, including 3D reconstruction, pathway planning, setting up localization marks and stent‐graft selection (see Figure 3). The DICOM files were manually imported into Endosize (Endosize Case Planning Software, Therenva SAS, Rennes, France), a commercially available imaging measurement software that automatically accomplished the extraction of anatomical data needed for endovascular repair. Then, Endosize offered pathway planning and stent‐graft selections.

Three‐dimensional reconstruction: patients completed CTA preoperatively, and the DICOM files were imported into Endosize to automatically generate a 3D reconstructed image of the aorta.

Pathway planning: three points were selected to depict the central line of the aorta: T1 (the origin of the suprarenal segment of the abdominal aorta) and T7/8 (bilateral femoral access position). All the T points were assisted points needed by Endosize and are not shown in Figure 3. According to the length of the automatically generated central line, the movement distance of the guide wire, catheter and stent graft is determined (see Figure 3).

Setting up localization marks: several points were set to assist the surgical robot in performing the surgeries (see Figure 3). These points were determined according to the surgeon's judgment. We set P1 as the target point of the wire tip and P2 as the target point of the catheter tip. We set up P3a and P3b to facilitate stent‐graft delivery and positioning. In addition, P4a and P4b were set to facilitate limb stent delivery and positioning.

Stent‐graft selection: another five points were selected to offer information on the length, diameter, angle and morphology of the aneurysm neck and the proximal landing zone of the stent, which were T2 (the point on the plane that is perpendicular to the central lumen line at the level of the lowest renal artery), T3 (the starting point of the aneurysm), T4 (the bifurcation of the abdominal aorta), and T5/6 (the starting point of the bilateral internal iliac arteries). Categories of stent grafts were determined according to the information above. After the surgeon chose the desired stent graft, related parameters were input into the parameter setting panel.

We set the intersection of the horizontal line of the lower edge of the fifth lumbar vertebra and the central line of the bilateral iliac arteries as 0 points—P_right_ (P_r_) or P_left_ (P_l_). After the surgeon loaded a new kind of endovascular device into the robotic system and manually pushed the tip of the device to 0 points (P_r_ or P_l_), the robot began the subsequent actions. This process was called initialization. It was performed by the surgeon in manual mode. Automatic robotic operation was started when endovascular devices were prepared at 0 points and finished when endovascular devices were retracted to 0 points.

### 2.5 In Vitro Evaluation Outcomes

**FIGURE 4:**
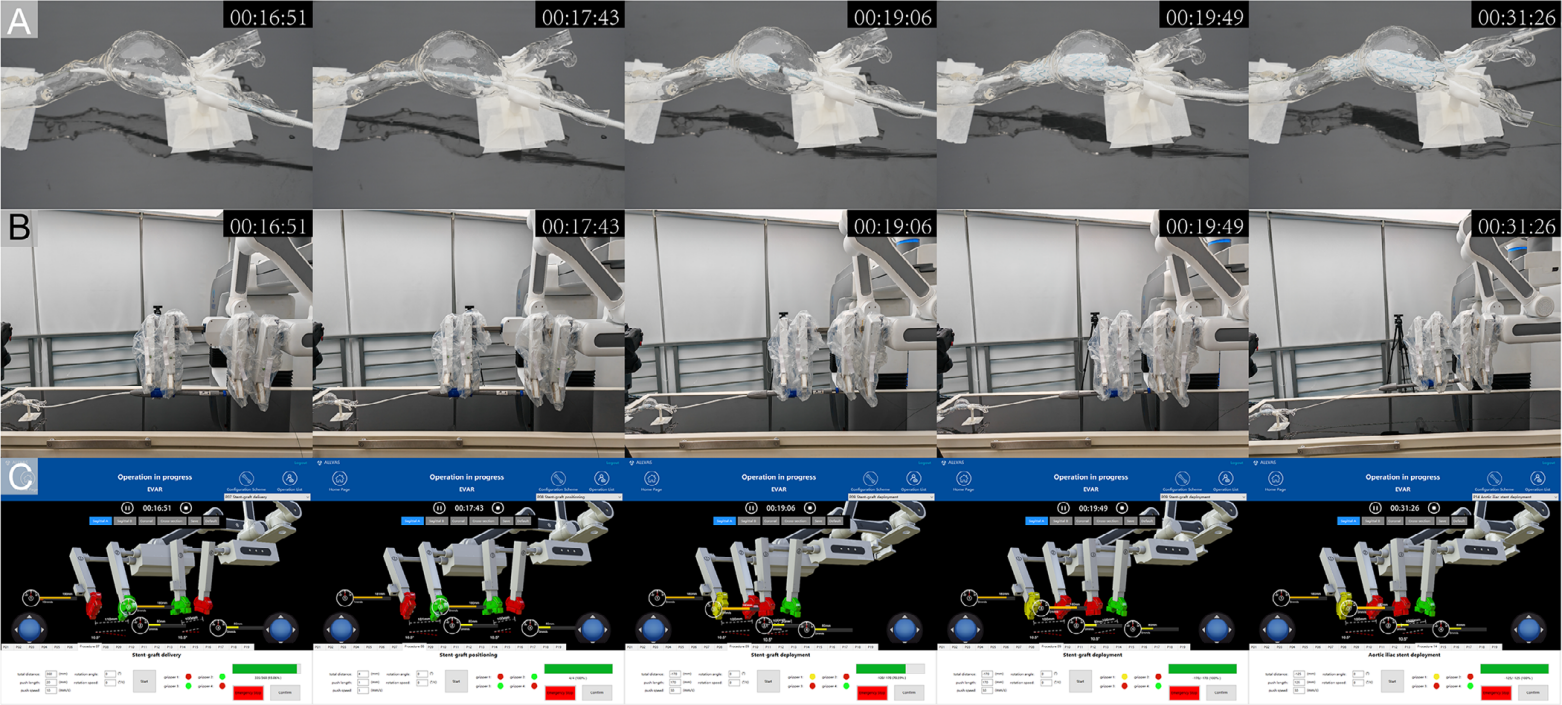
In vitro verification with 3D‐printed vascular models. The key steps of stent‐graft deployment are illustrated, including stent‐graft delivery, stent‐graft positioning, stent‐graft deployment, and limb stent deployment. This figure contains three views, namely, (A) a close view focusing on the vessel model, (B) a broad view showing the whole experimental environment, and (C) a screenshot of the monitor interface.

In vitro experiments were designed to evaluate feasibility prior to human application. The surgical algorithms of each model are illustrated in Table S2. Figure 4 depicts five distinct time points during the processes of automatic stent‐graft deployment performed on models and screenshots of the monitor, which achieved millimeter‐level precision in positioning. The initial time point (00:16:51) displays the phase of stent‐graft delivery, denoting the swift advancement of the stent graft as the seventh sequential step. The interface indicates that a distance of 360 mm is required to advance the stent graft in this phase. Currently, the stent graft has been advanced by 335 mm, with a push length of 20 mm and a push speed of 10 mm/s. The subsequent time point (00:17:43) showcases the status following the completion of stent‐graft positioning. The process of stent‐graft positioning involves the gradual advancement of the stent graft to achieve precise placement, constituting the eighth step in the procedure. The interface indicates that a 4 mm advancement is required in this step, with the stent graft currently having been advanced by 4 mm through successive 1 mm pushes at a rate of 1 mm/s. Subsequently, the deployment process of the stent graft, denoted as the nineth step, involves a withdrawal of 170 mm at a speed of 30 mm/s as indicated on the interface from the third (00:19:06) to the fourth (00:19:49) time point. The fifth time point (00:31:26) represents the conclusion of the limb stent deployment, marking the 14th step in the process. The interface indicates that at this stage, the limb stent can be retracted by 125 mm at a rate of 30 mm/s. Animated process was displayed in clip 2 of Video S1.

Outcomes of the automatic model group were compared with historical data of the hand‐controlled group from our previous study (see Table 2). In the hand‐controlled group, four patients underwent robotic‐assisted EVAR manually. Compared to manual robotic control, automatic procedures reduced mean operation time (128 ± 46 vs. 180 ± 41 min, *p* = 0.002) and fluoroscopy time (18 ± 6 vs. 24 ± 8 min, *p* = 0.003). Patient‐side radiation exposure was also lower in automatic model group (1188 ± 398 vs. 1546 ± 447 mGy, *p* = 0.001), while surgeon‐side exposure was comparable (4 ± 1 mGy). Technical success was achieved in all patients. The results indicated preliminary advantages of the automatic surgical algorithm in enhancing operation efficiency and reducing radiation exposure.

**TABLE 2:** Comparison of outcomes of the hand‐controlled group and automatic model group.

Hand‐controlled group					Automatic model group				
Items	Patient 1	Patient 2	Patient 3	Patient 4	Mean ± SD	Model 1	Model 2	Model 3	Model 4	Mean ± SD	*p*‐Value
Stents	Endurant	Endurant	Endurant	Endurant	—	Endurant	Endurant	Endurant	Endurant	—	—
Stent length (main body)	170	170	170	145	—	170	170	170	145	—	—
Number of stents	3	3	3	2	—	3	3	3	2	—	—
Operation time (min)	240	155	155	170	180 ± 41	193	91	99	127	128 ± 46	0.002
Total contrast volume used^a^ (mL)	170	130	110	110	130 ± 28	—	—	—	—	—	—
Fluoroscopy time (min)	35	21	20	19	24 ± 8	27	15	15	14	18 ± 6	0.003
Procedural radiation exposure (mGy)											
Surgeon side	5	4	4	3	4 ± 1	4	4	4	2	4 ± 1	0.182
Patient/model side	2200	1435	1350	1200	1546 ± 447	1765	1125	975	885	1188 ± 398	0.001
Technical success	Yes	Yes	Yes	Yes	—	Yes	Yes	Yes	Yes	—	—

*Note*: Data of the hand‐controlled group were from previous research [20].

^a^ Total contrast volume refers to the volume of contrast agent which is not only consumed for intravascular imaging but also used in the preparation of the angiographic catheter and high‐pressure injector tubing.

### 2.6 In Vivo Evaluation Outcomes

**FIGURE 5:**
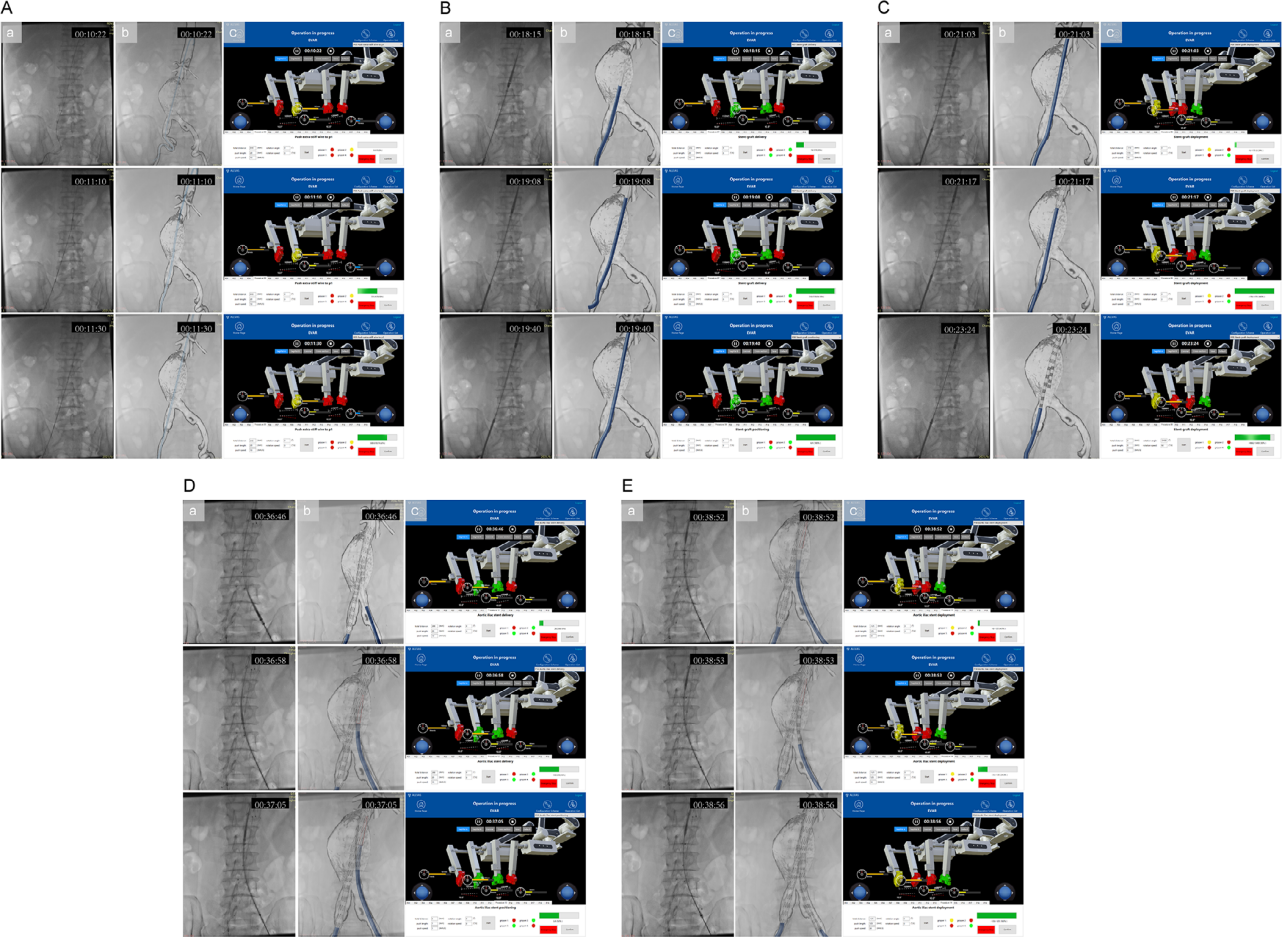
In vivo verification in enrolled patients. This figure displays five key steps in the endovascular aneurysm repair (EVAR) process, each accompanied by three types of images to demonstrate the sequential nature of each step, namely (a) the fluoroscopic view, (b) simulated view, and (c) monitor interface screenshot. (A) Advancement process of the super‐stiff wire (00:10:22‒00:11:30). (B) Rapid and slow advancement process of the stent graft (00:18:15‒00:19:40). (C) Deployment process of the stent graft (00:21:03‒00:23:24). (D) Rapid and slow advancement process of limb stent (00:36:46‒00:37:05). (E) Deployment process of limb stent (00:38:52‒00:38:56).

In vivo experiments involving four AAA patients was intended for further safety evaluation. According to personalized preoperative planning, customized surgical algorithms were programmed for each patient (see Table S3). The stent‐graft deployment processes of automatic robotic‐assisted EVAR performed on patients are illustrated in Figure 5. After the introduction of super‐stiff wire (see Figure 5A), the stent‐graft delivery system was first rapidly advanced to the vicinity of the target point at a speed of 10 mm/s, followed by slow advancement at 1 mm/s, ultimately achieving precise positioning at the target point (see Figure 5B). The stent graft was then deployed, followed by tip capture release process by counterclockwise rotating the tip capture release handle 5400° with a rotation speed of 90°/s (see Figure 5C). After manual contralateral limb gate cannulation and establishment of extra‐stiff wire channel, the limb stent was introduced and deployed in the same way as the main stent graft except for the tip capture release process (see Figure 5D,E).

The outcomes of the automatic patient group are illustrated in Table 3. In four AAA patients, automatic robotic EVAR achieved 100% technical and clinical success, with no intraoperative complications or major adverse cardiac events. The mean operation duration was 110 ± 47 min, with fluoroscopy time averaging 19 ± 6 min and patient‐side radiation exposure measuring 1251 ± 389 mGy. Contrast volume averaged 143 ± 43 mL, with 93 ± 28 mL used for intraoperative angiography. Stent grafts were deployed accurately at target positions, and postoperative CTA confirmed exclusion of aneurysms without endoleaks. Surgeon radiation exposure (4 ± 1 mGy) was consistent with in vitro findings, and no extra manual intervention was required during automatic steps.

**TABLE 3:** Outcomes of the automatic patient group.

Items	Patient A	Patient B	Patient C	Patient D	Mean ± SD
Stents	Endurant	Endurant	Endurant	Endurant	—
Stent length (main body)	170	170	170	170	—
Number of stents	2	2	2	3	—
Operation time (min)	178	75	82	104	110 ± 47
Blood loss (mL)	90	100	70	80	85 ± 13
Total contrast volume used^a^ (mL)	200	120	100	150	143 ± 43
Contrast volume used in intraoperative angiography (mL)	132	79	68	93	93 ± 28
Fluoroscopy time (min)	28	14	16	16	19 ± 6
Procedural radiation exposure (mGy)					
Surgeon side	3	3	4	4	4 ± 1
Patient side	1820	1005	1185	995	1251 ± 389
Technical success	Yes	Yes	Yes	Yes	—
Clinical success	Yes	Yes	Yes	Yes	—

^a^ Total contrast volume refers to the volume of contrast agent, which is not only consumed for intravascular imaging but also used in the preparation of the angiographic catheter and high‐pressure injector tubing.

## 3 Discussion

Automatic robotic‐assisted endovascular procedures represent a transformative evolution in vascular surgery, characterized by two foundational attributes: execution via an endovascular surgical robot and governance by pre‐programmed instructions. These features collectively enable a paradigm shift from operator‐dependent manual techniques toward standardized, reproducible, and precise interventions. In this study, we demonstrate that leveraging these attributes leads to three key advancements: optimized procedural workflows, enhanced precision in device manipulation, and homogenized surgical outcomes.

More optimized procedural workflows entail more optimized preoperative pathway planning and intraoperative movement planning. Our approach integrates preoperative pathway planning using Endosize, a validated imaging analysis platform that enables accurate determination of vascular landmarks and deployment zones. By defining precise start and end points for device navigation, we reduce reliance on intraoperative estimation—a known source of variability in stent positioning. The interpoint distance derived from such planning directly informs the linear movement parameters of the robotic system, thereby transforming anatomical data into executable motion commands. Furthermore, intraoperative movement planning is optimized through structured sequencing of device maneuvers and parameterization of motion (e.g., speed, direction, distance). This level of control is absent in conventional manual procedures and even in current teleoperated endovascular robotic systems such as the CorPath GRX, which offer limited automation beyond basic linear advancement. This approach is designed to promote the predictability and stability of surgical outcomes. Additionally, this optimization translated into tangible clinical benefits including reduced operation time and fluoroscopy exposure in both in vitro and in vivo settings, thereby reducing the workload for doctors and minimizing radiation exposure for patients. A similar conclusion has been reached with the CorPath GRX systems utilizing TechnIQ [25], further exemplifying how automatic procedures can enhance surgical efficiency.

More accurate device manipulation is reflected in stable mechanical control and quantification of each movement. The previous research has demonstrated that the novel endovascular surgical robot could control the advancement, retraction, and rotation of the manipulators with high accuracy [21, 26]. Building upon this capability, the system's movements have been further formalized into discrete and programmable motion parameters, encompassing linear displacement (including distance, direction, and velocity) and rotational motion (including angular displacement, direction, and rotational speed). The delivery of wires, catheters, and stent grafts only needs linear‐motion‐related parameters. For stent‐graft deployment, rotational‐motion‐related parameters are further needed. The status of movements can be adjusted precisely by adjusting the parameters. Combined with optimized preoperative planning, every device can be accurately delivered to an optimized targeted position, making the whole surgical process under precise control. Such a high degree of precision imposes stringent requirements on the robotic system's stability and reliability. Previous studies have reported an additional manual intervention rate ranging from 5.9% to 7.4% with the CorPath GRX system [27, 28]. Notably, the adoption of TechnIQ has been associated with an even higher intervention rate, exceeding 15% in certain cases [25]. The results of this study showed 100% technical and clinical success rates without additional manual intervention, demonstrating the reliability and robustness of the automatic surgical algorithm.

The two main characteristics mentioned above determine the homogeneity of surgical outcomes. One of the most compelling implications of surgical automation is the potential to decouple procedural outcomes from operator experience. In our in vivo experiments, four procedures were supervised by surgeons with varying levels of expertise, yet all achieved identical technical and clinical success, which suggests that the influence of the surgeon's level of surgical experience on procedural outcomes was substantially mitigated. These findings indicate that patients across hospitals of varying tiers could receive standardized, optimized, and precise surgical care, highlighting the potential of this technology to promote equitable access to high‐quality interventions [29].

Besides, less staff is exposed under the radiation environment compared to conventional technique, aligning with As Low As Reasonably Achievable (ALARA) principles in radiological safety [30]. Based on our experience, traditional surgical procedures typically involve a team of four staff members, consisting of one surgeon, two assistants, and one nurse. In contrast, robotic surgery necessitates a team of three individuals to manually operate the robotic system. The surgeon assumes the role of controlling the robot from the console, while one assistant is tasked with managing the loading and unloading of devices, and the nurse is responsible for transferring the devices. The distribution of responsibilities remains consistent whether the robot is operated manually or automatically. As a result, automatic robotic‐assisted surgery decreases the need for human resources in comparison to traditional surgical methods.

This study presents the first successful demonstration of automatic robotic‐assisted EVAR in both in vitro and in vivo evaluations, achieving 100% technical and clinical success with enhanced procedural optimization, precision, and outcome consistency. By anchoring automation to quantifiable parameters and validated imaging tools, we address core limitations in current robotic systems: fragmented automation, operator dependency, and lack of standardization. Our findings bridge a critical research gap between teleoperation and intelligent surgery in endovascular surgical robots. These results lay the groundwork for future clinical translation, offering a scalable framework for integrating robotic automation into complex vascular interventions.

Despite these advances, our study represents a proof‐of‐concept evaluation with a limited sample size, focusing primarily on feasibility and safety of the endovascular surgical robot with automatic surgical algorithm. While secondary outcomes showed favorable trends, larger studies are needed to confirm statistically significant benefits. Importantly, our current automation framework still relies on human‐defined planning inputs, leaving room for variability in preoperative decision making. To address this, future work will focus on AI‐assisted preoperative planning and further highlight the advantages of automatic surgery [31, 32, 33].

## 4 Materials and Methods

### 4.1 Study Design and Object

This study proposed an automatic surgical algorithm for endovascular surgical robot and conducted in vitro and in vivo experiments for feasibility and safety evaluation. A self‐designed master‐slave endovascular surgical robot manufactured by Aopeng Medical Co., Ltd. from Shanghai, China, was used as the algorithm deployment platform. The automatic surgical algorithm decomposed EVAR into discrete steps governed by parameters such as distance, speed, and rotational angle. Surgical steps were programmed via a touchscreen interface, enabling precise control of linear and rotational movements for devices.

For in vitro experiment, we verified the feasibility of the surgical algorithm and its radiation exposure and operation time with 3D printed vascular models, which were printed with a high transparency silicone material (Tiande Technology, Zhejiang, China) by a 3D printer using preoperative CTAs of four patients with AAA involved in our previous study [20]. The characteristics of the model materials are listed in Table S4. The inner walls of the models were lubricated in advance so that the stent graft could be introduced smoothly.

For in vivo experiment, a total of four patients diagnosed with AAA in our department were enrolled from February to April 2022. These patients underwent a 6‐month follow‐up post‐procedure. The criteria for participant inclusion and exclusion were in accordance with our prior research [20]. The in vivo study protocol received ethical approval from the Ethics Committee of Changhai Hospital (CHEC2021‐097), with informed consent secured from all participating patients. Additionally, the research project has been dutifully registered with the Chinese Clinical Trial Registry, bearing the registration number ChiCTR2400082087.

### 4.2 In Vitro Evaluation With Vascular Models

The procedures were deconstructed as described above, and the parameters of each procedure were extracted. Four sets of parameters of these patients were input into the surgical algorithm to perform EVAR on vascular models. Establishment of bilateral femoral accesses and general anesthesia were ostensibly performed on the models. Following the preparation of femoral artery access, the endovascular surgical robot was maneuvered to the operating table by the technician. The surgeon assumed responsibility for affixing the endovascular devices to the surgical robot and executing the initialization process, guided by the technician, ultimately achieving the docking procedure (clip 1 in Video S1 illustrates the docking process). Subsequently, upon the withdrawal of all endovascular devices by the surgical robot, the surgeon and technician collaboratively executed the undocking process. After the withdrawal of all endovascular devices, the access sites were managed using the Perclose ProGlide Suture‐Mediated Closure System (Abbott, Plymouth, MN, USA) and subsequently sutured to ensure secure closure. Fluoroscopy was implemented when endovascular devices were moved by the surgical robot. When measuring the radiation exposure on the surgeon side, the radiation dosimeter was placed on the console, which was behind the lead shield. When measuring the radiation exposure on the model side, the dosimeter was placed near the right femoral access which was about 30 cm away from the radiation source and covered with lead apron. Staff configuration is illustrated in Figure S1.

### 4.3 In Vivo Evaluation in AAA Patients

Surgical algorithms for EVAR were verified in 4 included patients with AAA. Surgical parameters for each patient were obtained through individual preoperative planning. For all patients, general anesthesia was given. Bilateral femoral accesses were achieved through percutaneous puncture, with the insertion of an 18F sheath to provide access for the guiding catheters and the stent‐graft system. The docking and undocking processes were completed in the same method as those in in vitro evaluation. Each procedure was monitored by doctors with various surgical experience, but preoperative planning was performed by an experienced doctor. All patients were prescribed appropriate medications postoperatively and followed up for 6 months with CTA examination.

### 4.4 Endpoints and Definition

The primary endpoint of our study is technical success, which is delineated as the successful deployment of the stent graft by the surgical robot without the necessity for additional manual intervention and in the absence of a type I or III endoleak. The secondary endpoints encompass the operation time, fluoroscopy time, and radiation exposure sustained by the surgeon, patients, and models across both in vitro and in vivo experiments. Operation time is hereby defined as the interval commencing from the setup of the robot to the completion of vascular suture. Furthermore, the contrast load was meticulously documented in the in vivo experiments. Clinical success is articulated as the successful deployment of the stent graft at the designated location, freedom from aneurysm‐related death, type I or III endoleak, graft infection or thrombosis, aneurysm dilation, aneurysm rupture, or conversion to open repair [34].

### 4.5 Statistical Analysis

Statistical analysis was performed using SPSS 27.0 (IBM, Armonk, NY, USA). For continuous data that were normally distributed such as age, values are presented as the mean ± standard deviation. For gender, disease history and other categorical data, values are presented as number and frequency (%). Comparisons of means between hand‐controlled group and automatic model group were made by paired *t*‐test. Statistical significance was set at *p* < 0.05.

## Author Contributions

B.L., C.S., and Q.L. designed the overall experiments and had unrestricted access to all data. B.L., C.S., S.X., and Q.L. performed the experiments. B.L. performed statistical analyses and prepared the first draft of the manuscript together with C.S. C.S. and Q.L. reviewed and edited the manuscript. C.S., L.Z., and W.G. contributed to the visualization work, including all the figures in the paper. Q.L. secured funding for the conduct of the study and publication of the paper. C.S. and K.W. contributed to the project administration. C.S., B.L., and Q.L. supervised the implementation of the study. All authors read and approved the final article and take responsibility for its content.

## Funding

This study was funded by the Program for Outstanding Academic Leader of Shanghai (23XD1405000) and the National Key Research and Development Program of China (2018AAA0102603). The funding sources were not involved in the design of the study and collection, analysis, and interpretation of data or in writing the manuscript.

## Ethics Statement

The in vivo study protocol was approved by the Ethics Committee of Changhai Hospital (CHEC2021‐097). Informed consent was obtained from all involved patients. The research project was officially registered with the Chinese Clinical Trial Registry under the registration number ChiCTR2400082087.

## Conflicts of Interest

Q.L. and K.W. are listed as inventors of patents on the presented endovascular surgical robot. The other authors declare that they have no competing financial interests.

## Supporting information




**Table S1**. Surgical algorithm for EVAR.
**Table S2**. Surgical algorithms for the automatic model group.
**Table S3**. Surgical algorithms for the automatic patient group.
**Table S4**. Material characteristics of the vascular phantom.
**Figure S1**. Staff configuration during automatic robotic‐assisted endovascular procedure.

**Video S1**. Animation of initial calibration and stent graft deployment.

## Data Availability

The data are available from the corresponding author upon reasonable request.
