# Supplementary material for: Unleashing the Potential: First‐in‐Human Evaluation of Automatic Robotic‐Assisted Endovascular Aortic Repair for Standardized Therapies
Source: MedComm (2020). 2025 Nov 18;6(12):e70489. doi: 10.1002/mco2.70489 (PMC12627237; doi:10.1002/mco2.70489)
Supplement: Supplementary file 1 — Table S1. Surgical algorithm for EVAR. Table S2. Surgical algorithms for the automatic model group. Table S3. Surgical algorithms for the automatic patient group. Table S4. Material characteristics of the vascular phantom. Figure S1. Staff configuration during automatic robotic‐assisted endovascular procedure. [file MCO2-6-e70489-s002.docx]

**Supplemental Information**

**Unleashing the Potential: First-in-Human Evaluation of Automatic Robotic-Assisted Endovascular Aortic Repair for Standardized Therapies**

Bowen Liang,^1,#^ Chao Song,^1,#^ Shibo Xia,^1^ Wenying Guo,^1^ Longtu Zhu,^1^ Kundong Wang,^2^ Qingsheng Lu^1,*^

^1^ Department of Vascular Surgery, Shanghai Changhai Hospital, Naval Medical University, Shanghai, 200433, China

^2^ Department of Instrument Science and Engineering, Shanghai Jiao Tong University, Shanghai, 200030, China

^#^ These two authors contributed equally to this work.

*Correspondence: luqs@newvascular.cn (Q.L.)

List of supplemental materials

Table S1. Surgical algorithm for EVAR.

Table S2. Surgical algorithms for the automatic model group.

Table S3. Surgical algorithms for the automatic patient group.

Table S4. Material characteristics of the vascular phantom.

Figure S1. Staff configuration during automatic robotic-assisted endovascular procedure.

Video S1. Animation of initial calibration and stent graft deployment.

Table S1. Surgical algorithm for EVAR.

| No. of the procedure | Procedure | Device | Movement distance  (mm) | Relative distance  (mm) | Push  speed  (mm/s) | Push  Length  (mm) | Rotation angle of M3 (°) | Rotation  Speed  （°/s） | Robotic arm involvement | M1 | M2 | M3 | M4 |
| --- | --- | --- | --- | --- | --- | --- | --- | --- | --- | --- | --- | --- | --- |
| 1 | Guidewire reaches right 0 point | Guidewire |  |  |  |  |  |  | N | clamp | hold | hold | hold |
|  | Push guidewire to P1 |  |  |  |  |  |  |  |  |  |  |  |  |
| 2 | Catheter reaches right 0 point | Pigtail catheter |  |  |  |  |  |  | N | clamp | hold | hold | clamp |
|  | Push catheter to P2 |  |  |  |  |  |  |  |  |  |  |  |  |
| 3 | Retract guidewire to right 0 point | Guidewire |  |  |  |  |  |  | N | clamp | hold | hold | clamp |
| 4 | Preoperative angiography | - | - | - | - | - | - | - | N | - | - | - | - |
| 5 | Extrastiff wire reaches right 0 point | Extrastiff wire |  |  |  |  |  |  | N | clamp | hold | clamp | clamp |
|  | Push extrastiff wire to P1 |  |  |  |  |  |  |  |  |  |  |  |  |
| 6 | Retract pigtail catheter to right 0 point | Pigtail catheter |  |  |  |  |  |  | A2 | loosen | loosen | clamp | clamp |
| 7 | Stent-graft reaches right 0 point | Stent-graft |  |  |  |  |  |  | N | clamp | loosen | loosen | clamp |
|  | Stent-graft delivery |  |  |  |  |  |  |  |  |  |  |  |  |
| 8 | Stent-graft positioning | Stent-graft |  |  |  |  |  |  | N | clamp | loosen | loosen | clamp |
| 9^a^ | Stent-graft deployment | Stent-graft |  |  |  |  |  |  | A1 | hold | clamp | clamp | loosen |
|  | Bare stent release |  |  |  |  |  |  |  |  |  |  |  |  |
| 10 | Delivery system withdrawal | Stent-graft |  |  |  |  |  |  | N | clamp | clamp | loosen | loosen |
| 11^b^ | Retract delivery system to right 0 point | Stent-graft |  |  |  |  |  |  | A2 | loosen | loosen | clamp | clamp |
|  |  |  |  |  |  |  |  |  |  |  |  |  |  |
| Establishment of extrastiff wire channel | | | | | | | | | | | | | |
| 12 | Limb stent reaches left 0 point | Limb stent |  |  |  |  |  |  | N | clamp | loosen | loosen | clamp |
|  | Limb stent delivery |  |  |  |  |  |  |  |  |  |  |  |  |
| 13 | Limb stent positioning | Limb stent |  |  |  |  |  |  | N | clamp | loosen | loosen | clamp |
| 14^a^ | Limb stent deployment | Limb stent |  |  |  |  |  |  | A1 | hold | clamp | clamp | loosen |
| 15 | Delivery system withdrawal | Limb stent |  |  |  |  |  |  | N | clamp | clamp | loosen | loosen |
| 16^b^ | Retract delivery system to left 0 point | Limb stent |  |  |  |  |  |  | A2 | loosen | loosen | clamp | clamp |
|  |  |  |  |  |  |  |  |  |  |  |  |  |  |
| 17 | Pigtail catheter reaches left 0 point | Pigtail catheter |  |  |  |  |  |  | N | clamp | hold | hold | clamp |
|  | Push pigtail catheter to P2 |  |  |  |  |  |  |  |  |  |  |  |  |
| 18 | Retract extrastiff wire to left 0 point | Extrastiff wire |  |  |  |  |  |  | N | clamp | hold | hold | clamp |
| 19 | Postoperative angiography | - | - | - | - | - | - | - | N | - | - | - | - |

In the column of movement distance, the value indicates the movement distance of endovascular devices. A plus sign indicates advancement, and a minus sign indicates withdrawal. In the column of relative distance, the value means the distance from the tip of the device to the 0 point at the end of the step. In the column of push speed, the value means the speed of executing a single push. In the column of push length, the value means the distance of a single push. In the column of the rotation angle of M3, the value means the rotation angle of M3 when releasing the bare stent, and a plus sign means clockwise, and a minus sign means counterclockwise. In some steps, robotic arms are also involved. N stands for No. A stands for Arm that is involved in the step. ^a^ The delivery system remained stationary, and the movement distance represents the retraction distance of the slider. ^b^ Retraction of delivery system was divided into two parts. The upper line represents rapid retraction, and the lower line represents slow retraction. Steps 12-16 can be repeated if an extender is needed.

Table S2. Surgical algorithms for automatic model group.

Model 1

| No. of the procedure | Procedure | Device | Movement distance  (mm) | Relative distance  (mm) | Push  speed  (mm/s) | Push  Length  (mm) | Rotation angle of M3 (°) | Rotation  Speed  （°/s） | Robotic arm involvement | M1 | M2 | M3 | M4 |  |  |  |
| --- | --- | --- | --- | --- | --- | --- | --- | --- | --- | --- | --- | --- | --- | --- | --- | --- |
| 1 | Guidewire reaches right 0 point | Guidewire | +700 | 700 | 10 | 20 | - | - | N | clamp | hold | hold | hold |  |  |  |
|  | Push guidewire to P1 |  |  |  |  |  |  |  |  |  |  |  |  |  |  |  |
| 2 | Catheter reaches right 0 point | Pigtail catheter | +680 | 680 | 10 | 20 | - | - | N | clamp | hold | hold | clamp |  |  |  |
|  | Push catheter to P2 |  |  |  |  |  |  |  |  |  |  |  |  |  |  |  |
| 3 | Retract guidewire to right 0 pint | Guidewire | -700 | 0 | 10 | 20 | - | - | N | clamp | hold | hold | clamp |  |  |  |
| 4 | Preoperative angiography | - | - | - | - | - | - | - | N | - | - | - | - |  |  |  |
| 5 | Extra-stiff wire reaches right 0 point | Extra-stiff wire | +700 | 700 | 10 | 20 | - | - | N | clamp | hold | clamp | clamp |  |  |  |
|  | Push extra-stiff wire to P1 |  |  |  |  |  |  |  |  |  |  |  |  |  |  |  |
| 6 | Retract pigtail catheter to right 0 point | Pigtail catheter | -680 | 0 | 10 | 20 | - | - | A2 | loosen | loosen | clamp | clamp |  |  |  |
| 7 | Stent-graft reaches right 0 point | Stent-graft | +360 | 360 | 10 | 20 | - | - | N | clamp | loosen | loosen | clamp |  |  |  |
|  | Stent-graft delivery |  |  |  |  |  |  |  |  |  |  |  |  |  |  |  |
| 8 | Stent-graft positioning | Stent-graft | +4 | 364 | 1 | 1 | - | - | N | clamp | loosen | loosen | clamp |  |  |  |
| 9^a^ | Stent-graft deployment | Stent-graft | -170 | 364 | 30 | 170 | - | - | A1 | hold | clamp | clamp | loosen |  |  |  |
|  | Bare stent release |  | - | 364 | - | - | -5,400 | 90 |  |  |  |  |  |  |  |  |
| 10 | Delivery system withdrawal | Stent-graft | -170 | 194 | 10 | 170 | - | - | N | clamp | clamp | loosen | loosen |  |  |  |
| 11^b^ | Retract delivery system to right 0 point | Stent-graft | -180 | 14 | 10 | 20 | - | - | A2 | loosen | loosen | clamp | clamp |  |  |  |
|  |  |  | -14 | 0 | 1 | 1 |  |  |  |  |  |  |  |  |  |  |
| Establishment of extra-stiff wire channel | | | | | | | | | | | | | |  |  | Establishment of extra-stiff wire channel |
| 12 | Limb stent reaches left 0 point | Limb stent | +220(+160) | 220(160) | 10 | 20 | - | - | N | clamp | loosen | loosen | clamp |  |  |  |
|  | Limb stent delivery |  |  |  |  |  |  |  |  |  |  |  |  |  |  |  |
| 13 | Limb stent positioning | Limb stent | +6 | 226(166) | 1 | 1 | - | - | N | clamp | loosen | loosen | clamp |  |  |  |
| 14^a^ | Limb stent deployment | Limb stent | -125 | 226(166) | 30 | 125 | - | - | A1 | hold | clamp | clamp | loosen |  |  |  |
| 15 | Delivery system withdrawal | Limb stent | -125 | 101(41) | 10 | 125 | - | - | N | clamp | clamp | loosen | loosen |  |  |  |
| 16^b^ | Retract delivery system to left 0 point | Limb stent | -100(-40) | 1(1) | 10 | 20 | - | - | A2 | loosen | loosen | clamp | clamp |  |  |  |
|  |  |  | -1(-1) | 0(0) | 1 | 1 |  |  |  |  |  |  |  |  |  |  |
| 17 | Pigtail catheter reaches left 0 point | Pigtail catheter | +680 | 680 | 10 | 20 | - | - | N | clamp | hold | hold | clamp |  |  |  |
|  | Push pigtail catheter to P2 |  |  |  |  |  |  |  |  |  |  |  |  |  |  |  |
| 18 | Retract extra-stiff wire to left 0 point | Extra-stiff wire | -700 | 0 | 10 | 20 | - | - | N | clamp | hold | hold | clamp |  |  |  |
| 19 | Postoperative angiography | - | - | - | - | - | - | - | N | - | - | - | - |  |  |  |

In the column of movement distance, the value indicates the movement distance of endovascular devices. A plus sign indicates advancement, and a minus sign indicates withdrawal. In the column of relative distance, the value means the distance from the tip of the device to the 0 point at the end of the step. In the column of push speed, the value means the speed of executing a single push. In the column of push length, the value means the distance of a single push. In the column of the rotation angle of M3, the value means the rotation angle of M3 when releasing the bare stent, and a plus sign means clockwise, and a minus sign means counterclockwise. In some steps, robotic arms are also involved. N stands for No. A stands for Arm that is involved in the step. ^a^ The delivery system remained stationary, and the movement distance represents the retraction distance of the slider. ^b^ Retraction of delivery system was divided into two parts. The upper line represents rapid retraction, and the lower line represents slow retraction. An extender was used in this case and step 12-16 were repeated. Corresponding parameters were showed in brackets.

Model 2

| No. of the procedure | Procedure | Device | Movement distance  (mm) | Relative distance  (mm) | Push  speed  (mm/s) | Push  Length  (mm) | Rotation angle of M3 (°) | Rotation  Speed  （°/s） | Robotic arm involvement | M1 | M2 | M3 | M4 |  |  |  |  |  |
| --- | --- | --- | --- | --- | --- | --- | --- | --- | --- | --- | --- | --- | --- | --- | --- | --- | --- | --- |
| 1 | Guidewire reaches right 0 point | Guidewire | +820 | 820 | 10 | 20 | - | - | N | clamp | hold | hold | hold |  |  |  |  |  |
|  | Push guidewire to P1 |  |  |  |  |  |  |  |  |  |  |  |  |  |  |  |  |  |
| 2 | Catheter reaches right 0 point | Pigtail catheter | +800 | 800 | 10 | 20 | - | - | N | clamp | hold | hold | clamp |  |  |  |  |  |
|  | Push catheter to P2 |  |  |  |  |  |  |  |  |  |  |  |  |  |  |  |  |  |
| 3 | Retract guidewire to right 0 pint | Guidewire | -820 | 0 | 10 | 20 | - | - | N | clamp | hold | hold | clamp |  |  |  |  |  |
| 4 | Preoperative angiography | - | - | - | - | - | - | - | N | - | - | - | - |  |  |  |  |  |
| 5 | Extra-stiff wire reaches right 0 point | Extra-stiff wire | +820 | 820 | 10 | 20 | - | - | N | clamp | hold | clamp | clamp |  |  |  |  |  |
|  | Push extra-stiff wire to P1 |  |  |  |  |  |  |  |  |  |  |  |  |  |  |  |  |  |
| 6 | Retract pigtail catheter to right 0 point | Pigtail catheter | -800 | 0 | 10 | 20 | - | - | A2 | loosen | loosen | clamp | clamp |  |  |  |  |  |
| 7 | Stent-graft reaches right 0 point | Stent-graft | +420 | 420 | 10 | 20 | - | - | N | clamp | loosen | loosen | clamp |  |  |  |  |  |
|  | Stent-graft delivery |  |  |  |  |  |  |  |  |  |  |  |  |  |  |  |  |  |
| 8 | Stent-graft positioning | Stent-graft | +4 | 424 | 1 | 1 | - | - | N | clamp | loosen | loosen | clamp |  |  |  |  |  |
| 9^a^ | Stent-graft deployment | Stent-graft | -170 | 424 | 30 | 170 | - | - | A1 | hold | clamp | clamp | loosen |  |  |  |  |  |
|  | Bare stent release |  | - | 424 | - | - | -5,400 | 90 |  |  |  |  |  |  |  |  |  |  |
| 10 | Delivery system withdrawal | Stent-graft | -170 | 254 | 10 | 170 | - | - | N | clamp | clamp | loosen | loosen |  |  |  |  |  |
| 11^b^ | Retract delivery system to right 0 point | Stent-graft | -240 | 14 | 10 | 20 | - | - | A2 | loosen | loosen | clamp | clamp |  |  |  |  |  |
|  |  |  | -14 | 0 | 1 | 1 |  |  |  |  |  |  |  |  |  |  |  |  |
| Establishment of extra-stiff wire channel | | | | | | | | | | | | | |  |  |  |  | Establishment of extra-stiff wire channel |
| 12 | Limb stent reaches left 0 point | Limb stent | +260(+190) | 260(190) | 10 | 20 | - | - | N | clamp | loosen | loosen | clamp |  |  |  |  |  |
|  | Limb stent delivery |  |  |  |  |  |  |  |  |  |  |  |  |  |  |  |  |  |
| 13 | Limb stent positioning | Limb stent | +6 | 266(196) | 1 | 1 | - | - | N | clamp | loosen | loosen | clamp |  |  |  |  |  |
| 14^a^ | Limb stent deployment | Limb stent | -125 | 266(196) | 30 | 125 | - | - | A1 | hold | clamp | clamp | loosen |  |  |  |  |  |
| 15 | Delivery system withdrawal | Limb stent | -125 | 141(71) | 10 | 125 | - | - | N | clamp | clamp | loosen | loosen |  |  |  |  |  |
| 16^b^ | Retract delivery system to left 0 point | Limb stent | -140(-60) | 1(11) | 10 | 20 | - | - | A2 | loosen | loosen | clamp | clamp |  |  |  |  |  |
|  |  |  | -1(-11) | 0(0) | 1 | 1 |  |  |  |  |  |  |  |  |  |  |  |  |
| 17 | Pigtail catheter reaches left 0 point | Pigtail catheter | +800 | 800 | 10 | 20 | - | - | N | clamp | hold | hold | clamp |  |  |  |  |  |
|  | Push pigtail catheter to P2 |  |  |  |  |  |  |  |  |  |  |  |  |  |  |  |  |  |
| 18 | Retract extra-stiff wire to left 0 point | Extra-stiff wire | -820 | 0 | 10 | 20 | - | - | N | clamp | hold | hold | clamp |  |  |  |  |  |
| 19 | Postoperative angiography | - | - | - | - | - | - | - | N | - | - | - | - |  |  |  |  |  |

In the column of movement distance, the value indicates the movement distance of endovascular devices. A plus sign indicates advancement, and a minus sign indicates withdrawal. In the column of relative distance, the value means the distance from the tip of the device to the 0 point at the end of the step. In the column of push speed, the value means the speed of executing a single push. In the column of push length, the value means the distance of a single push. In the column of the rotation angle of M3, the value means the rotation angle of M3 when releasing the bare stent, and a plus sign means clockwise, and a minus sign means counterclockwise. In some steps, robotic arms are also involved. N stands for No. A stands for Arm that is involved in the step. ^a^ The delivery system remained stationary, and the movement distance represents the retraction distance of the slider. ^b^ Retraction of delivery system was divided into two parts. The upper line represents rapid retraction, and the lower line represents slow retraction. An extender was used in this case and step 12-16 were repeated. Corresponding parameters were showed in brackets.

Model 3

| No. of the procedure | Procedure | Device | Movement distance  (mm) | Relative distance  (mm) | Push  speed  (mm/s) | Push  Length  (mm) | Rotation angle of M3 (°) | Rotation  Speed  （°/s） | Robotic arm involvement | M1 | M2 | M3 | M4 |  |  |  |  |  |
| --- | --- | --- | --- | --- | --- | --- | --- | --- | --- | --- | --- | --- | --- | --- | --- | --- | --- | --- |
| 1 | Guidewire reaches right 0 point | Guidewire | +730 | 730 | 10 | 20 | - | - | N | clamp | hold | hold | hold |  |  |  |  |  |
|  | Push guidewire to P1 |  |  |  |  |  |  |  |  |  |  |  |  |  |  |  |  |  |
| 2 | Catheter reaches right 0 point | Pigtail catheter | +710 | 710 | 10 | 20 | - | - | N | clamp | hold | hold | clamp |  |  |  |  |  |
|  | Push catheter to P2 |  |  |  |  |  |  |  |  |  |  |  |  |  |  |  |  |  |
| 3 | Retract guidewire to right 0 pint | Guidewire | -730 | 0 | 10 | 20 | - | - | N | clamp | hold | hold | clamp |  |  |  |  |  |
| 4 | Preoperative angiography | - | - | - | - | - | - | - | N | - | - | - | - |  |  |  |  |  |
| 5 | Extra-stiff wire reaches right 0 point | Extra-stiff wire | +730 | 730 | 10 | 20 | - | - | N | clamp | hold | clamp | clamp |  |  |  |  |  |
|  | Push extra-stiff wire to P1 |  |  |  |  |  |  |  |  |  |  |  |  |  |  |  |  |  |
| 6 | Retract pigtail catheter to right 0 point | Pigtail catheter | --710 | 0 | 10 | 20 | - | - | A2 | loosen | loosen | clamp | clamp |  |  |  |  |  |
| 7 | Stent-graft reaches right 0 point | Stent-graft | +400 | 400 | 10 | 20 | - | - | N | clamp | loosen | loosen | clamp |  |  |  |  |  |
|  | Stent-graft delivery |  |  |  |  |  |  |  |  |  |  |  |  |  |  |  |  |  |
| 8 | Stent-graft positioning | Stent-graft | +4 | 404 | 1 | 1 | - | - | N | clamp | loosen | loosen | clamp |  |  |  |  |  |
| 9^a^ | Stent-graft deployment | Stent-graft | -170 | 404 | 30 | 170 | - | - | A1 | hold | clamp | clamp | loosen |  |  |  |  |  |
|  | Bare stent release |  | - | 404 | - | - | -5,400 | 90 |  |  |  |  |  |  |  |  |  |  |
| 10 | Delivery system withdrawal | Stent-graft | -170 | 234 | 10 | 170 | - | - | N | clamp | clamp | loosen | loosen |  |  |  |  |  |
| 11^b^ | Retract delivery system to right 0 point | Stent-graft | -220 | 14 | 10 | 20 | - | - | A2 | loosen | loosen | clamp | clamp |  |  |  |  |  |
|  |  |  | -14 | 0 | 1 | 1 |  |  |  |  |  |  |  |  |  |  |  |  |
| Establishment of extra-stiff wire channel | | | | | | | | | | | | | |  |  |  |  | Establishment of extra-stiff wire channel |
| 12 | Limb stent reaches left 0 point | Limb stent | +230(+200) | 230(200) | 10 | 20 | - | - | N | clamp | loosen | loosen | clamp |  |  |  |  |  |
|  | Limb stent delivery |  |  |  |  |  |  |  |  |  |  |  |  |  |  |  |  |  |
| 13 | Limb stent positioning | Limb stent | 6 | 236(206) | 1 | 1 | - | - | N | clamp | loosen | loosen | clamp |  |  |  |  |  |
| 14^a^ | Limb stent deployment | Limb stent | -125 | 236(206) | 30 | 125 | - | - | A1 | hold | clamp | clamp | loosen |  |  |  |  |  |
| 15 | Delivery system withdrawal | Limb stent | -125 | 111(81) | 10 | 125 | - | - | N | clamp | clamp | loosen | loosen |  |  |  |  |  |
| 16^b^ | Retract delivery system to left 0 point | Limb stent | -100(-80) | 11(1) | 10 | 20 | - | - | A2 | loosen | loosen | clamp | clamp |  |  |  |  |  |
|  |  |  | -11(-1) | 0(0) | 1 | 1 |  |  |  |  |  |  |  |  |  |  |  |  |
| 17 | Pigtail catheter reaches left 0 point | Pigtail catheter | +710 | 710 | 10 | 20 | - | - | N | clamp | hold | hold | clamp |  |  |  |  |  |
|  | Push pigtail catheter to P2 |  |  |  |  |  |  |  |  |  |  |  |  |  |  |  |  |  |
| 18 | Retract extra-stiff wire to left 0 point | Extra-stiff wire | -730 | 0 | 10 | 20 | - | - | N | clamp | hold | hold | clamp |  |  |  |  |  |
| 19 | Postoperative angiography | - | - | - | - | - | - | - | N | - | - | - | - |  |  |  |  |  |

In the column of movement distance, the value indicates the movement distance of endovascular devices. A plus sign indicates advancement, and a minus sign indicates withdrawal. In the column of relative distance, the value means the distance from the tip of the device to the 0 point at the end of the step. In the column of push speed, the value means the speed of executing a single push. In the column of push length, the value means the distance of a single push. In the column of the rotation angle of M3, the value means the rotation angle of M3 when releasing the bare stent, and a plus sign means clockwise, and a minus sign means counterclockwise. In some steps, robotic arms are also involved. N stands for No. A stands for Arm that is involved in the step. ^a^ The delivery system remained stationary, and the movement distance represents the retraction distance of the slider. ^b^ Retraction of delivery system was divided into two parts. The upper line represents rapid retraction, and the lower line represents slow retraction.An extender was used in this case and step 12-16 were repeated. Corresponding parameters were showed in brackets.

Model 4

| No. of the procedure | Procedure | Device | Movement distance  (mm) | Relative distance  (mm) | Push  speed  (mm/s) | Push  Length  (mm) | Rotation angle of M3 (°) | Rotation  Speed  （°/s） | Robotic arm involvement | M1 | M2 | M3 | M4 |  |  |  |  |  |
| --- | --- | --- | --- | --- | --- | --- | --- | --- | --- | --- | --- | --- | --- | --- | --- | --- | --- | --- |
| 1 | Guidewire reaches right 0 point | Guidewire | +750 | 750 | 10 | 20 | - | - | N | clamp | hold | hold | hold |  |  |  |  |  |
|  | Push guidewire to P1 |  |  |  |  |  |  |  |  |  |  |  |  |  |  |  |  |  |
| 2 | Catheter reaches right 0 point | Pigtail catheter | +730 | 730 | 10 | 20 | - | - | N | clamp | hold | hold | clamp |  |  |  |  |  |
|  | Push catheter to P2 |  |  |  |  |  |  |  |  |  |  |  |  |  |  |  |  |  |
| 3 | Retract guidewire to right 0 pint | Guidewire | -750 | 0 | 10 | 20 | - | - | N | clamp | hold | hold | clamp |  |  |  |  |  |
| 4 | Preoperative angiography | - | - | - | - | - | - | - | N | - | - | - | - |  |  |  |  |  |
| 5 | Extra-stiff wire reaches right 0 point | Extra-stiff wire | +750 | 750 | 10 | 20 | - | - | N | clamp | hold | clamp | clamp |  |  |  |  |  |
|  | Push extra-stiff wire to P1 |  |  |  |  |  |  |  |  |  |  |  |  |  |  |  |  |  |
| 6 | Retract pigtail catheter to right 0 point | Pigtail catheter | -730 | 0 | 10 | 20 | - | - | A2 | loosen | loosen | clamp | clamp |  |  |  |  |  |
| 7 | Stent-graft reaches right 0 point | Stent-graft | +410 | 410 | 10 | 20 | - | - | N | clamp | loosen | loosen | clamp |  |  |  |  |  |
|  | Stent-graft delivery |  |  |  |  |  |  |  |  |  |  |  |  |  |  |  |  |  |
| 8 | Stent-graft positioning | Stent-graft | +4 | 414 | 1 | 1 | - | - | N | clamp | loosen | loosen | clamp |  |  |  |  |  |
| 9^a^ | Stent-graft deployment | Stent-graft | -145 | 414 | 30 | 170 | - | - | A1 | hold | clamp | clamp | loosen |  |  |  |  |  |
|  | Bare stent release |  | - | 414 | - | - | -5,400 | 90 |  |  |  |  |  |  |  |  |  |  |
| 10 | Delivery system withdrawal | Stent-graft | -145 | 269 | 10 | 170 | - | - | N | clamp | clamp | loosen | loosen |  |  |  |  |  |
| 11^b^ | Retract delivery system to right 0 point | Stent-graft | -260 | 9 | 10 | 20 | - | - | A2 | loosen | loosen | clamp | clamp |  |  |  |  |  |
|  |  |  | -9 | 0 | 1 | 1 |  |  |  |  |  |  |  |  |  |  |  |  |
| Establishment of extra-stiff wire channel | | | | | | | | | | | | | |  |  |  |  | Establishment of extra-stiff wire channel |
| 12 | Limb stent reaches left 0 point | Limb stent | +250 | 250 | 10 | 20 | - | - | N | clamp | loosen | loosen | clamp |  |  |  |  |  |
|  | Limb stent delivery |  |  |  |  |  |  |  |  |  |  |  |  |  |  |  |  |  |
| 13 | Limb stent positioning | Limb stent | +6 | 256 | 1 | 1 | - | - | N | clamp | loosen | loosen | clamp |  |  |  |  |  |
| 14^a^ | Limb stent deployment | Limb stent | -125 | 256 | 30 | 125 | - | - | A1 | hold | clamp | clamp | loosen |  |  |  |  |  |
| 15 | Delivery system withdrawal | Limb stent | -125 | 131 | 10 | 125 | - | - | N | clamp | clamp | loosen | loosen |  |  |  |  |  |
| 16^b^ | Retract delivery system to left 0 point | Limb stent | -120 | 11 | 10 | 20 | - | - | A2 | loosen | loosen | clamp | clamp |  |  |  |  |  |
|  |  |  | -11 | 0 | 1 | 1 |  |  |  |  |  |  |  |  |  |  |  |  |
| 17 | Pigtail catheter reaches left 0 point | Pigtail catheter | +730 | 730 | 10 | 20 | - | - | N | clamp | hold | hold | clamp |  |  |  |  |  |
|  | Push pigtail catheter to P2 |  |  |  |  |  |  |  |  |  |  |  |  |  |  |  |  |  |
| 18 | Retract extra-stiff wire to left 0 point | Extra-stiff wire | -750 | 0 | 10 | 20 | - | - | N | clamp | hold | hold | clamp |  |  |  |  |  |
| 19 | Postoperative angiography | - | - | - | - | - | - | - | N | - | - | - | - |  |  |  |  |  |

In the column of movement distance, the value indicates the movement distance of endovascular devices. A plus sign indicates advancement, and a minus sign indicates withdrawal. In the column of relative distance, the value means the distance from the tip of the device to the 0 point at the end of the step. In the column of push speed, the value means the speed of executing a single push. In the column of push length, the value means the distance of a single push. In the column of the rotation angle of M3, the value means the rotation angle of M3 when releasing the bare stent, and a plus sign means clockwise, and a minus sign means counterclockwise. In some steps, robotic arms are also involved. N stands for No. A stands for Arm that is involved in the step. ^a^ The delivery system remained stationary, and the movement distance represents the retraction distance of the slider. ^b^ Retraction of delivery system was divided into two parts. The upper line represents rapid retraction, and the lower line represents slow retraction.

Table S3. Surgical algorithms for automatic patient group.

Patient 1

| No. of the procedure | Procedure | Device | Movement distance  (mm) | Relative distance  (mm) | Push  speed  (mm/s) | Push  Length  (mm) | Rotation angle of M3 (°) | Rotation  Speed  （°/s） | Robotic arm involvement | M1 | M2 | M3 | M4 |  |  |  |
| --- | --- | --- | --- | --- | --- | --- | --- | --- | --- | --- | --- | --- | --- | --- | --- | --- |
| 1 | Guidewire reaches right 0 point | Guidewire | +670 | 670 | 10 | 20 | - | - | N | clamp | hold | hold | hold |  |  |  |
|  | Push guidewire to P1 |  |  |  |  |  |  |  |  |  |  |  |  |  |  |  |
| 2 | Catheter reaches right 0 point | Pigtail catheter | +650 | 650 | 10 | 20 | - | - | N | clamp | hold | hold | clamp |  |  |  |
|  | Push catheter to P2 |  |  |  |  |  |  |  |  |  |  |  |  |  |  |  |
| 3 | Retract guidewire to right 0 pint | Guidewire | -670 | 0 | 10 | 20 | - | - | N | clamp | hold | hold | clamp |  |  |  |
| 4 | Preoperative angiography | - | - | - | - | - | - | - | N | - | - | - | - |  |  |  |
| 5 | Extra-stiff wire reaches right 0 point | Extra-stiff wire | +670 | 670 | 10 | 20 | - | - | N | clamp | hold | clamp | clamp |  |  |  |
|  | Push extra-stiff wire to P1 |  |  |  |  |  |  |  |  |  |  |  |  |  |  |  |
| 6 | Retract pigtail catheter to right 0 point | Pigtail catheter | -650 | 0 | 10 | 20 | - | - | A2 | loosen | loosen | clamp | clamp |  |  |  |
| 7 | Stent-graft reaches right 0 point | Stent-graft | +370 | 370 | 10 | 20 | - | - | N | clamp | loosen | loosen | clamp |  |  |  |
|  | Stent-graft delivery |  |  |  |  |  |  |  |  |  |  |  |  |  |  |  |
| 8 | Stent-graft positioning | Stent-graft | +4 | 374 | 1 | 1 | - | - | N | clamp | loosen | loosen | clamp |  |  |  |
| 9^a^ | Stent-graft deployment | Stent-graft | -170 | 374 | 30 | 170 | - | - | A1 | hold | clamp | clamp | loosen |  |  |  |
|  | Bare stent release |  | - | 374 | - | - | -5,400 | 90 |  |  |  |  |  |  |  |  |
| 10 | Delivery system withdrawal | Stent-graft | -170 | 204 | 10 | 170 | - | - | N | clamp | clamp | loosen | loosen |  |  |  |
| 11^b^ | Retract delivery system to right 0 point | Stent-graft | -200 | 4 | 10 | 20 | - | - | A2 | loosen | loosen | clamp | clamp |  |  |  |
|  |  |  | -4 | 0 | 1 | 1 |  |  |  |  |  |  |  |  |  |  |
| Establishment of extra-stiff wire channel | | | | | | | | | | | | | |  |  | Establishment of extra-stiff wire channel |
| 12 | Limb stent reaches left 0 point | Limb stent | +200 | 200 | 10 | 20 | - | - | N | clamp | loosen | loosen | clamp |  |  |  |
|  | Limb stent delivery |  |  |  |  |  |  |  |  |  |  |  |  |  |  |  |
| 13 | Limb stent positioning | Limb stent | 6 | 206 | 1 | 1 | - | - | N | clamp | loosen | loosen | clamp |  |  |  |
| 14^a^ | Limb stent deployment | Limb stent | -125 | 206 | 30 | 125 | - | - | A1 | hold | clamp | clamp | loosen |  |  |  |
| 15 | Delivery system withdrawal | Limb stent | -125 | 181 | 10 | 125 | - | - | A1 | clamp | clamp | loosen | loosen |  |  |  |
| 16^b^ | Retract delivery system to left 0 point | Limb stent | -180 | 1 | 10 | 20 | - | - | A2 | loosen | loosen | clamp | clamp |  |  |  |
|  |  |  | -1 | 0 | 1 | 1 |  |  |  |  |  |  |  |  |  |  |
| 17 | Pigtail catheter reaches left 0 point | Pigtail catheter | +650 | 650 | 10 | 20 | - | - | N | clamp | hold | hold | clamp |  |  |  |
|  | Push pigtail catheter to P2 |  |  |  |  |  |  |  |  |  |  |  |  |  |  |  |
| 18 | Retract extra-stiff wire to left 0 point | Extra-stiff wire | -670 | 0 | 10 | 20 | - | - | N | clamp | hold | hold | clamp |  |  |  |
| 19 | Postoperative angiography | - | - | - | - | - | - | - | N | - | - | - | - |  |  |  |

In the column of movement distance, the value indicates the movement distance of endovascular devices. A plus sign indicates advancement, and a minus sign indicates withdrawal. In the column of relative distance, the value means the distance from the tip of the device to the 0 point at the end of the step. In the column of push speed, the value means the speed of executing a single push. In the column of push length, the value means the distance of a single push. In the column of the rotation angle of M3, the value means the rotation angle of M3 when releasing the bare stent, and a plus sign means clockwise, and a minus sign means counterclockwise. In some steps, robotic arms are also involved. N stands for No. A stands for Arm that is involved in the step. ^a^ The delivery system remained stationary, and the movement distance represents the retraction distance of the slider. ^b^ Retraction of delivery system was divided into two parts. The upper line represents rapid retraction, and the lower line represents slow retraction.

Patient 2

| No. of the procedure | Procedure | Device | Movement distance  (mm) | Relative distance  (mm) | Push  speed  (mm/s) | Push  Length  (mm) | Rotation angle of M3 (°) | Rotation  Speed  （°/s） | Robotic arm involvement | M1 | M2 | M3 | M4 |  |  |  |  |  |
| --- | --- | --- | --- | --- | --- | --- | --- | --- | --- | --- | --- | --- | --- | --- | --- | --- | --- | --- |
| 1 | Guidewire reaches right 0 point | Guidewire | +690 | 690 | 10 | 20 | - | - | N | clamp | hold | hold | hold |  |  |  |  |  |
|  | Push guidewire to P1 |  |  |  |  |  |  |  |  |  |  |  |  |  |  |  |  |  |
| 2 | Catheter reaches right 0 point | Pigtail catheter | +670 | 670 | 10 | 20 | - | - | N | clamp | hold | hold | clamp |  |  |  |  |  |
|  | Push catheter to P2 |  |  |  |  |  |  |  |  |  |  |  |  |  |  |  |  |  |
| 3 | Retract guidewire to right 0 pint | Guidewire | -690 | 0 | 10 | 20 | - | - | N | clamp | hold | hold | clamp |  |  |  |  |  |
| 4 | Preoperative angiography | - | - | - | - | - | - | - | N | - | - | - | - |  |  |  |  |  |
| 5 | Extra-stiff wire reaches right 0 point | Extra-stiff wire | +690 | 690 | 10 | 20 | - | - | N | clamp | hold | clamp | clamp |  |  |  |  |  |
|  | Push extra-stiff wire to P1 |  |  |  |  |  |  |  |  |  |  |  |  |  |  |  |  |  |
| 6 | Retract pigtail catheter to right 0 point | Pigtail catheter | -670 | 0 | 10 | 20 | - | - | A2 | loosen | loosen | clamp | clamp |  |  |  |  |  |
| 7 | Stent-graft reaches right 0 point | Stent-graft | +430 | 430 | 10 | 20 | - | - | N | clamp | loosen | loosen | clamp |  |  |  |  |  |
|  | Stent-graft delivery |  |  |  |  |  |  |  |  |  |  |  |  |  |  |  |  |  |
| 8 | Stent-graft positioning | Stent-graft | +4 | 434 | 1 | 1 | - | - | N | clamp | loosen | loosen | clamp |  |  |  |  |  |
| 9^a^ | Stent-graft deployment | Stent-graft | -170 | 434 | 30 | 170 | - | - | A1 | hold | clamp | clamp | loosen |  |  |  |  |  |
|  | Bare stent release |  | - | 434 | - | - | -5,400 | 90 |  |  |  |  |  |  |  |  |  |  |
| 10 | Delivery system withdrawal | Stent-graft | -170 | 264 | 10 | 170 | - | - | N | clamp | clamp | loosen | loosen |  |  |  |  |  |
| 11^b^ | Retract delivery system to right 0 point | Stent-graft | -260 | 4 | 10 | 20 | - | - | A2 | loosen | loosen | clamp | clamp |  |  |  |  |  |
|  |  |  | -4 | 0 | 1 | 1 |  |  |  |  |  |  |  |  |  |  |  |  |
| Establishment of extra-stiff wire channel | | | | | | | | | | | | | |  |  |  |  | Establishment of extra-stiff wire channel |
| 12 | Limb stent reaches left 0 point | Limb stent | +220 | 220 | 10 | 20 | - | - | N | clamp | loosen | loosen | clamp |  |  |  |  |  |
|  | Limb stent delivery |  |  |  |  |  |  |  |  |  |  |  |  |  |  |  |  |  |
| 13 | Limb stent positioning | Limb stent | +6 | 226 | 1 | 1 | - | - | N | clamp | loosen | loosen | clamp |  |  |  |  |  |
| 14^a^ | Limb stent deployment | Limb stent | -125 | 226 | 30 | 125 | - | - | A1 | hold | clamp | clamp | loosen |  |  |  |  |  |
| 15 | Delivery system withdrawal | Limb stent | -125 | 101 | 10 | 125 | - | - | A1 | clamp | clamp | loosen | loosen |  |  |  |  |  |
| 16^b^ | Retract delivery system to left 0 point | Limb stent | -100 | 1 | 10 | 20 | - | - | A2 | loosen | loosen | clamp | clamp |  |  |  |  |  |
|  |  |  | -1 | 0 | 1 | 1 |  |  |  |  |  |  |  |  |  |  |  |  |
| 17 | Pigtail catheter reaches left 0 point | Pigtail catheter | +670 | 670 | 10 | 20 | - | - | N | clamp | hold | hold | clamp |  |  |  |  |  |
|  | Push pigtail catheter to P2 |  |  |  |  |  |  |  |  |  |  |  |  |  |  |  |  |  |
| 18 | Retract extra-stiff wire to left 0 point | Extra-stiff wire | -690 | 0 | 10 | 20 | - | - | N | clamp | hold | hold | clamp |  |  |  |  |  |
| 19 | Postoperative angiography | - | - | - | - | - | - | - | N | - | - | - | - |  |  |  |  |  |

In the column of movement distance, the value indicates the movement distance of endovascular devices. A plus sign indicates advancement, and a minus sign indicates withdrawal. In the column of relative distance, the value means the distance from the tip of the device to the 0 point at the end of the step. In the column of push speed, the value means the speed of executing a single push. In the column of push length, the value means the distance of a single push. In the column of the rotation angle of M3, the value means the rotation angle of M3 when releasing the bare stent, and a plus sign means clockwise, and a minus sign means counterclockwise. In some steps, robotic arms are also involved. N stands for No. A stands for Arm that is involved in the step. ^a^ The delivery system remained stationary, and the movement distance represents the retraction distance of the slider. ^b^ Retraction of delivery system was divided into two parts. The upper line represents rapid retraction, and the lower line represents slow retraction.

Patient 3

| No. of the procedure | Procedure | Device | Movement distance  (mm) | Relative distance  (mm) | Push  speed  (mm/s) | Push  Length  (mm) | Rotation angle of M3 (°) | Rotation  Speed  （°/s） | Robotic arm involvement | M1 | M2 | M3 | M4 |  |  |  |  |  |
| --- | --- | --- | --- | --- | --- | --- | --- | --- | --- | --- | --- | --- | --- | --- | --- | --- | --- | --- |
| 1 | Guidewire reaches right 0 point | Guidewire | +800 | 800 | 10 | 20 | - | - | N | clamp | hold | hold | hold |  |  |  |  |  |
|  | Push guidewire to P1 |  |  |  |  |  |  |  |  |  |  |  |  |  |  |  |  |  |
| 2 | Catheter reaches right 0 point | Pigtail catheter | +780 | 780 | 10 | 20 | - | - | N | clamp | hold | hold | clamp |  |  |  |  |  |
|  | Push catheter to P2 |  |  |  |  |  |  |  |  |  |  |  |  |  |  |  |  |  |
| 3 | Retract guidewire to right 0 pint | Guidewire | -800 | 0 | 10 | 20 | - | - | N | clamp | hold | hold | clamp |  |  |  |  |  |
| 4 | Preoperative angiography | - | - | - | - | - | - | - | N | - | - | - | - |  |  |  |  |  |
| 5 | Extra-stiff wire reaches right 0 point | Extra-stiff wire | +800 | 800 | 10 | 20 | - | - | N | clamp | hold | clamp | clamp |  |  |  |  |  |
|  | Push extra-stiff wire to P1 |  |  |  |  |  |  |  |  |  |  |  |  |  |  |  |  |  |
| 6 | Retract pigtail catheter to right 0 point | Pigtail catheter | -780 | 0 | 10 | 20 | - | - | A2 | loosen | loosen | clamp | clamp |  |  |  |  |  |
| 7 | Stent-graft reaches right 0 point | Stent-graft | +470 | 470 | 10 | 20 | - | - | N | clamp | loosen | loosen | clamp |  |  |  |  |  |
|  | Stent-graft delivery |  |  |  |  |  |  |  |  |  |  |  |  |  |  |  |  |  |
| 8 | Stent-graft positioning | Stent-graft | +4 | 474 | 1 | 1 | - | - | N | clamp | loosen | loosen | clamp |  |  |  |  |  |
| 9^a^ | Stent-graft deployment | Stent-graft | -170 | 474 | 30 | 170 | - | - | A1 | hold | clamp | clamp | loosen |  |  |  |  |  |
|  | Bare stent release |  | - | 474 | - | - | -5,400 | 90 |  |  |  |  |  |  |  |  |  |  |
| 10 | Delivery system withdrawal | Stent-graft | -170 | 304 | 10 | 170 | - | - | N | clamp | clamp | loosen | loosen |  |  |  |  |  |
| 11^b^ | Retract delivery system to right 0 point | Stent-graft | -300 | 4 | 10 | 20 | - | - | A2 | loosen | loosen | clamp | clamp |  |  |  |  |  |
|  |  |  | -4 | 0 | 1 | 1 |  |  |  |  |  |  |  |  |  |  |  |  |
| Establishment of extra-stiff wire channel | | | | | | | | | | | | | |  |  |  |  | Establishment of extra-stiff wire channel |
| 12 | Limb stent reaches left 0 point | Limb stent | +300 | 300 | 10 | 20 | - | - | N | clamp | loosen | loosen | clamp |  |  |  |  |  |
|  | Limb stent delivery |  |  |  |  |  |  |  |  |  |  |  |  |  |  |  |  |  |
| 13 | Limb stent positioning | Limb stent | +6 | 306 | 1 | 1 | - | - | N | clamp | loosen | loosen | clamp |  |  |  |  |  |
| 14^a^ | Limb stent deployment | Limb stent | -125 | 306 | 30 | 125 | - | - | A1 | hold | clamp | clamp | loosen |  |  |  |  |  |
| 15 | Delivery system withdrawal | Limb stent | -125 | 181 | 10 | 125 | - | - | N | clamp | clamp | loosen | loosen |  |  |  |  |  |
| 16^b^ | Retract delivery system to left 0 point | Limb stent | -180 | 1 | 10 | 20 | - | - | A2 | loosen | loosen | clamp | clamp |  |  |  |  |  |
|  |  |  | -1 | 0 | 1 | 1 |  |  |  |  |  |  |  |  |  |  |  |  |
| 17 | Pigtail catheter reaches left 0 point | Pigtail catheter | +780 | 780 | 10 | 20 | - | - | N | clamp | hold | hold | clamp |  |  |  |  |  |
|  | Push pigtail catheter to P2 |  |  |  |  |  |  |  |  |  |  |  |  |  |  |  |  |  |
| 18 | Retract extra-stiff wire to left 0 point | Extra-stiff wire | -800 | 0 | 10 | 20 | - | - | N | clamp | hold | hold | clamp |  |  |  |  |  |
| 19 | Postoperative angiography | - | - | - | - | - | - | - | N | - | - | - | - |  |  |  |  |  |

In the column of movement distance, the value indicates the movement distance of endovascular devices. A plus sign indicates advancement, and a minus sign indicates withdrawal. In the column of relative distance, the value means the distance from the tip of the device to the 0 point at the end of the step. In the column of push speed, the value means the speed of executing a single push. In the column of push length, the value means the distance of a single push. In the column of the rotation angle of M3, the value means the rotation angle of M3 when releasing the bare stent, and a plus sign means clockwise, and a minus sign means counterclockwise. In some steps, robotic arms are also involved. N stands for No. A stands for Arm that is involved in the step. ^a^ The delivery system remained stationary, and the movement distance represents the retraction distance of the slider. ^b^ Retraction of delivery system was divided into two parts. The upper line represents rapid retraction, and the lower line represents slow retraction.

Patient 4

| No. of the procedure | Procedure | Device | Movement distance  (mm) | Relative distance  (mm) | Push  speed  (mm/s) | Push  Length  (mm) | Rotation angle of M3 (°) | Rotation  Speed  （°/s） | Robotic arm involvement | M1 | M2 | M3 | M4 |  |  |  |  |  |
| --- | --- | --- | --- | --- | --- | --- | --- | --- | --- | --- | --- | --- | --- | --- | --- | --- | --- | --- |
| 1 | Guidewire reaches right 0 point | Guidewire | +740 | 740 | 10 | 20 | - | - | N | clamp | hold | hold | hold |  |  |  |  |  |
|  | Push guidewire to P1 |  |  |  |  |  |  |  |  |  |  |  |  |  |  |  |  |  |
| 2 | Catheter reaches right 0 point | Pigtail catheter | +720 | 720 | 10 | 20 | - | - | N | clamp | hold | hold | clamp |  |  |  |  |  |
|  | Push catheter to P2 |  |  |  |  |  |  |  |  |  |  |  |  |  |  |  |  |  |
| 3 | Retract guidewire to right 0 pint | Guidewire | -740 | 0 | 10 | 20 | - | - | N | clamp | hold | hold | clamp |  |  |  |  |  |
| 4 | Preoperative angiography | - | - | - | - | - | - | - | N | - | - | - | - |  |  |  |  |  |
| 5 | Extra-stiff wire reaches right 0 point | Extra-stiff wire | +740 | 740 | 10 | 20 | - | - | N | clamp | hold | clamp | clamp |  |  |  |  |  |
|  | Push extra-stiff wire to P1 |  |  |  |  |  |  |  |  |  |  |  |  |  |  |  |  |  |
| 6 | Retract pigtail catheter to right 0 point | Pigtail catheter | +720 | 0 | 10 | 20 | - | - | A2 | loosen | loosen | clamp | clamp |  |  |  |  |  |
| 7 | Stent-graft reaches right 0 point | Stent-graft | +380 | 380 | 10 | 20 | - | - | N | clamp | loosen | loosen | clamp |  |  |  |  |  |
|  | Stent-graft delivery |  |  |  |  |  |  |  |  |  |  |  |  |  |  |  |  |  |
| 8 | Stent-graft positioning | Stent-graft | +4 | 384 | 1 | 1 | - | - | N | clamp | loosen | loosen | clamp |  |  |  |  |  |
| 9^a^ | Stent-graft deployment | Stent-graft | -170 | 384 | 30 | 170 | - | - | A1 | hold | clamp | clamp | loosen |  |  |  |  |  |
|  | Bare stent release |  | - | 384 | - | - | -5,400 | 90 |  |  |  |  |  |  |  |  |  |  |
| 10 | Delivery system withdrawal | Stent-graft | -170 | 214 | 10 | 170 | - | - | N | clamp | clamp | loosen | loosen |  |  |  |  |  |
| 11^b^ | Retract delivery system to right 0 point | Stent-graft | -200 | 14 | 10 | 20 | - | - | A2 | loosen | loosen | clamp | clamp |  |  |  |  |  |
|  |  |  | -14 | 0 | 1 | 1 |  |  |  |  |  |  |  |  |  |  |  |  |
| Establishment of extra-stiff wire channel | | | | | | | | | | | | | |  |  |  |  | Establishment of extra-stiff wire channel |
| 12 | Limb stent reaches left 0 point | Limb stent | +280(+220) | 280(220) | 10 | 20 | - | - | N | clamp | loosen | loosen | clamp |  |  |  |  |  |
|  | Limb stent delivery |  |  |  |  |  |  |  |  |  |  |  |  |  |  |  |  |  |
| 13 | Limb stent positioning | Limb stent | +6 | 286(226) | 1 | 1 | - | - | N | clamp | loosen | loosen | clamp |  |  |  |  |  |
| 14^a^ | Limb stent deployment | Limb stent | -125 | 286(226) | 30 | 125 | - | - | A1 | hold | clamp | clamp | loosen |  |  |  |  |  |
| 15 | Delivery system withdrawal | Limb stent | -125 | 161(101) | 10 | 125 | - | - | N | clamp | clamp | loosen | loosen |  |  |  |  |  |
| 16^b^ | Retract delivery system to left 0 point | Limb stent | -160(-100) | 1(1) | 10 | 20 | - | - | A2 | loosen | loosen | clamp | clamp |  |  |  |  |  |
|  |  |  | -1(-1) | 0(0) | 1 | 1 |  |  |  |  |  |  |  |  |  |  |  |  |
| 17 | Pigtail catheter reaches left 0 point | Pigtail catheter | +720 | 720 | 10 | 20 | - | - | N | clamp | hold | hold | clamp |  |  |  |  |  |
|  | Push pigtail catheter to P2 |  |  |  |  |  |  |  |  |  |  |  |  |  |  |  |  |  |
| 18 | Retract extra-stiff wire to left 0 point | Extra-stiff wire | -740 | 0 | 10 | 20 | - | - | N | clamp | hold | hold | clamp |  |  |  |  |  |
| 19 | Postoperative angiography | - | - | - | - | - | - | - | N | - | - | - | - |  |  |  |  |  |

In the column of movement distance, the value indicates the movement distance of endovascular devices. A plus sign indicates advancement, and a minus sign indicates withdrawal. In the column of relative distance, the value means the distance from the tip of the device to the 0 point at the end of the step. In the column of push speed, the value means the speed of executing a single push. In the column of push length, the value means the distance of a single push. In the column of the rotation angle of M3, the value means the rotation angle of M3 when releasing the bare stent, and a plus sign means clockwise, and a minus sign means counterclockwise. In some steps, robotic arms are also involved. N stands for No. A stands for Arm that is involved in the step. ^a^ The delivery system remained stationary, and the movement distance represents the retraction distance of the slider. ^b^ Retraction of delivery system was divided into two parts. The upper line represents rapid retraction, and the lower line represents slow retraction. An extender was used in this case and step 12-16 were repeated. Corresponding parameters were showed in brackets.

Table S4. Material characteristics of the vascular phantom.

| Item | Parameters |
| --- | --- |
| Specification | TD-9150-50 |
| Appearance | Transparent |
| Hardness (Shore A) | 48-50 |
| Density(g/cm^3^) | 1.13 |
| Tensilestrength (Mpa) | ≥9 |
| Elongation (%) | ≥500 |
| Tensile set (%) | 18-22 |
| Tear strength (KN/m) | ≥25 |
| Linear shrinkage | 3.2-3.6 |
| Curing system | After the A/B components are mixed according to the weight ratio of 1:1, they are formed by 150mm×150mm×2mm at 180℃/2min |

Figure S1. Staff configuration during automatic robotic-assisted endovascular procedure.
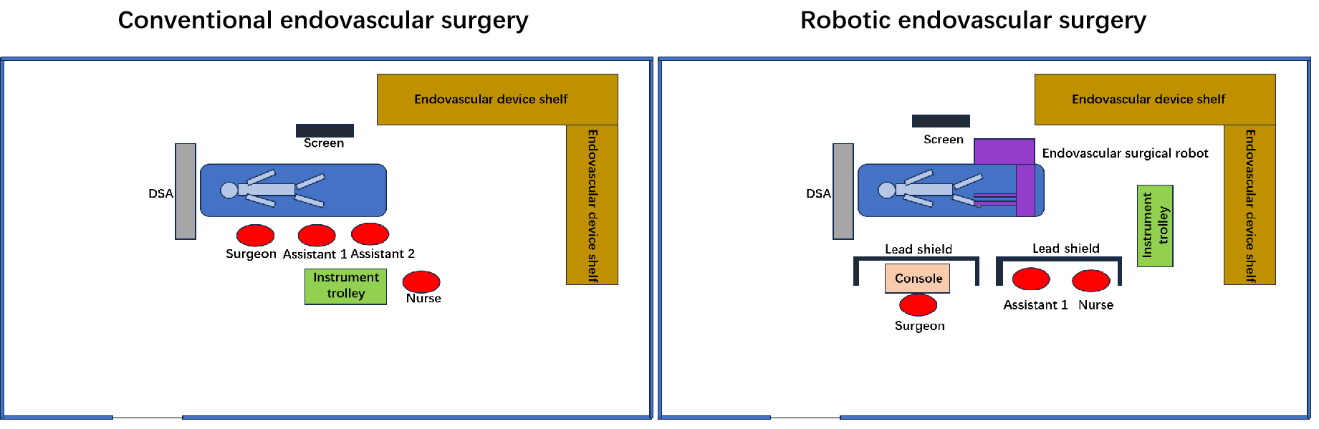


Video S1. Animation of initial calibration and stent graft deployment.
